# Utility of blue-light cystoscopy and tumor-informed ctDNA for management of recurrent high-risk NMIBC with prostatic urethral involvement: A case report

**DOI:** 10.1016/j.eucr.2026.103362

**Published:** 2026-01-27

**Authors:** Mahdi Hemmati Ghavshough, Melinda Z. Fu, Jennifer Sykes, Disha Patel, Saum Ghodoussipour, Vignesh T. Packiam

**Affiliations:** Department of Urology, Rutgers Cancer Institute, New Brunswick, NJ, USA

**Keywords:** Urinary bladder neoplasms, Urethra, Cystoscopy, DNA, Neoplasm, Minimal residual disease, Circulating tumor DNA

## Abstract

Non–muscle-invasive bladder cancer (NMIBC) with prostatic urethral involvement (PUI) is rare and difficult to manage, especially after intravesical therapy or pelvic radiation. We report a 78-year-old man with recurrent high-risk NMIBC and PUI after bacillus Calmette–Guérin and mitomycin C. Blue-light cystoscopy (BLC) during restaging identified additional carcinoma in situ and high-grade T1 disease not seen with white light. Histopathology showed no stromal invasion, and tumor-informed circulating tumor DNA (ctDNA) testing was negative, indicating no molecular residual disease. Despite favorable findings, the patient elected radical cystectomy, which revealed no residual tumor. This case highlights the complementary roles of BLC and ctDNA.

## Highlights

• Blue-light cystoscopy improved detection of CIS and high-grade lesions in recurrent NMIBC.
• Tumor-informed ctDNA (Signatera™) provided molecular confirmation of low residual disease.
• Combined use of BLC and ctDNA enhanced staging accuracy in NMIBC with prostatic urethral involvement.
• Multimodal evaluation guided individualized management in a very high-risk, BCG-unresponsive patient.
• Radical cystectomy pathology confirmed no residual carcinoma despite complex disease features.

## 1 Introduction

Non–muscle-invasive bladder cancer (NMIBC) accounts for approximately 70 % of bladder cancers and is commonly treated with TURBT followed by intravesical therapy; however, recurrence and progression remain frequent in high-risk, BCG-unresponsive disease.^1^ Prostatic urethral involvement (PUI) represents a particularly challenging scenario, as the prostatic urethra may serve as a sanctuary site for malignant cells, increasing the risk of understaging and adverse oncologic outcomes.^1^ Accurate restaging is therefore critical. Blue-light cystoscopy (BLC) with intravesical hexaminolevulinate improves detection of residual and flat lesions, including CIS, compared with white-light cystoscopy, and is guideline-endorsed for high-risk NMIBC.^2^ However, BLC is limited to visual assessment and cannot detect minimal residual disease. Circulating tumor DNA (ctDNA) has emerged as a sensitive biomarker for disease monitoring, with tumor-informed assays such as Signatera™ enabling detection of molecular residual disease through patient-specific genomic profiling.^3^ While BLC and ctDNA have each shown utility in bladder cancer management, their combined application in NMIBC with PUI has not been previously reported. We present a case demonstrating how integration of BLC-guided TURBT and tumor-informed ctDNA informed staging accuracy and surgical decision-making in recurrent high-risk NMIBC with PUI.

## 2 Case presentation

A 78-year-old male with history of prostate and bladder cancer presented to the urology clinic with recurrent painless gross hematuria. He denied suprapubic or flank pain, dysuria, fever, weight loss, or bone pain. His past medical history was significant for prostate adenocarcinoma diagnosed in 2014, treated with androgen deprivation therapy and external beam radiation therapy. He also had a history of urethral stricture managed with clean intermittent catheterization and active Crohn's disease controlled with mesalamine. He was a retired sheet metal worker and a former smoker (four pack-years, quit in 1970) with no family history of urologic malignancies.

Non-contrast computed tomography (CT) of the abdomen and pelvis demonstrated a 2.3 cm soft tissue mass along the right bladder wall. Urinalysis revealed 3–5 red blood cells per high-power field, and laboratory parameters were within normal limits, including a hemoglobin level of 14.4 g/dL and a serum creatinine of 0.85 mg/dL. Diagnostic cystoscopy and TURBT confirmed multifocal high-grade Ta and T1 urothelial carcinoma with uninvolved muscularis propria. There was separate high-grade Ta cancer in the prostatic urethra.

The patient completed induction intravesical BCG therapy, which was complicated by bladder spasms and leakage during initial instillations. He received 1 cycle of maintenance BCG, but this was subsequently discontinued due to recurrent urinary tract infections. His 6-month surveillance cystoscopy in revealed recurrence, for which six weekly instillations of intravesical mitomycin C were administered in the community. Surveillance cystoscopy was negative until the following year when recurrence was visualized. TURBT demonstrated recurrent high-grade Ta lesions at the bladder dome and high-grade Ta and focal high-grade T1 disease in the prostatic urethra. Based on AUA/SUO risk stratification—including prior BCG failure, PUI, and prior pelvic radiation—the patient was categorized as very high risk.

**Fig. 1:**
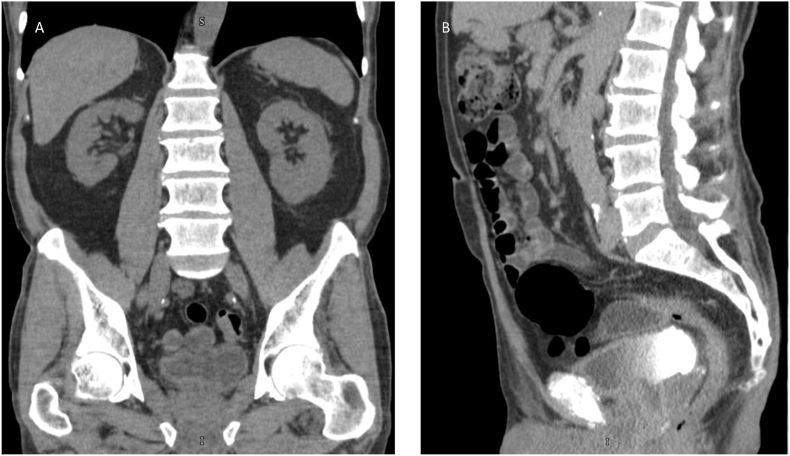
CT urography demonstrating mild concentric bladder wall thickening without discrete lesions or evidence of metastatic disease. (A) Axial contrast-enhanced CT image of the pelvis showing mild concentric thickening of the urinary bladder wall without focal intraluminal lesions. (B) Coronal reformatted contrast-enhanced CT image confirming uniform bladder wall thickening with no radiographic evidence of locally advanced or metastatic disease.

The patient was referred to our institution and a re-staging TURBT under BLC was performed, identifying lesions within the prostatic urethra and confirming no residual disease at the bladder dome or left posterior wall. Pathology revealed CIS involving von Brunn's nests and high-grade papillary urothelial carcinoma with focal subepithelial stromal invasion (pT1) but no deep stromal or glandular involvement. CT urography demonstrated mild concentric bladder wall thickening without discrete lesions or metastases (Fig. 1).

Tumor-informed ctDNA testing using the Signatera™ assay was negative. Management options were discussed with the patient including bladder-sparing therapy with or without transurethral resection of prostate, or radical cystoprostatectomy. Important anatomic considerations included his history of radiation and Crohns disease requiring immunomodulation. His BLC and Signatera findings were favorable, supporting bladder-sparing therapy. However, due to his ongoing immunosuppression and patients wish to fully optimize oncologic outcomes, the decision was made to proceed with radical cystectomy with cutaneous ureterostomy for definitive management. Final pathology demonstrated no residual carcinoma in the bladder, prostatic urethra, or lymph nodes.

## 3 Discussion

NMIBC generally carries a favorable prognosis when managed with TURBT followed by intravesical therapy; however, recurrence and progression remain common among high-risk patients, particularly those unresponsive to BCG therapy.^1^ PUI represents a particularly high-risk feature, as malignant cells within the prostatic ducts may evade exposure to intravesical agents.^4^ Accurate staging of PUI is challenging, because microscopic stromal invasion or nodal involvement may remain undetected until evaluation of radical cystoprostatectomy specimens.^4^ This potential for understaging contributes to inferior oncologic outcomes. In the present case, the coexistence of PUI, prior pelvic radiation, Crohn's disease, and BCG-unresponsive high-risk NMIBC necessitated meticulous restaging and an individualized management approach.

Enhanced cystoscopy has emerged as an important adjunct to conventional WLC in the management of NMIBC. BLC using intravesical hexaminolevulinate improves detection of residual and flat lesions, particularly CIS.^5^ BLC-guided TURBT has been associated with reduced recurrence rates and is endorsed by the AUA, EAU, and NCCN, especially for patients with high-risk disease or suspected CIS.^5^ The AUA/SUO guideline recommends offering BLC at the time of TURBT, when available, to enhance tumor detection and decrease recurrence (Moderate Recommendation; Evidence Strength: Grade B).^5^ In this case, BLC identified lesions not visible under WLC, allowing more complete resection and improved staging accuracy. This emphasizes the clinical utility of BLC in reducing residual disease and minimizing the risk of understaging in complex NMIBC presentations. BLC has been shown to have increasing utility in patients who have received numerous prior courses of intravesical.^6^

According to the NCCN Guidelines (Version 3.2024), radical cystectomy remains the preferred treatment for patients with high-risk NMIBC who are BCG-unresponsive or intolerant Although bladder-preserving approaches may be considered in select nonsurgical candidates, cystectomy remains the oncologic standard^7^. Therefore, in AUA very high-risk patients, enhanced diagnostic tools such as BLC may improve staging confidence but are unlikely to change guideline-directed recommendations for definitive surgery^7^. Despite this, BLC retains clinical utility in reducing residual disease and minimizing the risk of understaging, particularly in complex NMIBC presentations. Its diagnostic benefit may be especially pronounced in patients who have undergone multiple prior courses of intravesical therapy.^6^

Despite its increased sensitivity, BLC is associated with a recognized risk of false-positive findings, particularly in the setting of inflammation, recent intravesical therapy, especially BCG, and recent TURBT. Inflammatory changes can alter urothelial fluorescence patterns, reducing diagnostic specificity.^8^ In this patient, underlying Crohn's disease and repeated intravesical treatments increased the likelihood of false-positive BLC findings, necessitating cautious interpretation. Accordingly, while BLC improves detection of lesions—especially CIS—fluorescence alone is not diagnostic, and histopathologic confirmation remains essential. BLC should therefore be regarded as a complementary staging tool that enhances visualization and resection completeness rather than a standalone determinant of disease burden.

In this case, BLC refined staging accuracy and supported shared decision-making but did not alter guideline-directed management. When combined with negative tumor-informed ctDNA findings, it provided reassurance against occult disease; however, given the patient's multiple high-risk features, proceeding with radical cystectomy remained consistent with established guidelines.

ctDNA has emerged as a promising biomarker for cancer detection, treatment monitoring, and prognostication, offering a minimally invasive method to track tumor dynamics in real time and frequently detect recurrence earlier than radiologic imaging.^9^ Detection of ctDNA following curative-intent therapy represents molecular residual disease (MRD), which correlates strongly with recurrence and reduced disease-free survival across multiple malignancies. Tumor-informed ctDNA assays, customized to a patient's individual tumor mutations, have demonstrated high sensitivity and specificity for MRD detection.^10^

Serial ctDNA testing in the adjuvant setting has shown sensitivities ranging from 80 to 100 % and specificities from 88 to 100 %.^10^ The positive predictive value remains consistently high, while the negative predictive value improves with longitudinal monitoring.^10^ Notably, postoperative ctDNA testing as early as 2–4 weeks may help identify patients who could benefit from intensified adjuvant therapy.^10^ Signatera™, a tumor-informed 16-plex PCR assay, has been shown to detect molecular relapse in urothelial carcinoma a median of 96 days before radiographic recurrence.^11^ In a real-world NMIBC cohort, ctDNA positivity was observed in 35 % of patients and frequently prompted earlier clinical intervention.^12^

In this patient, the combination of negative ctDNA findings and complete BLC-guided resection suggested a low risk of residual disease, supporting consideration of a bladder-sparing strategy. However, given additional clinical factors—including prior radiation exposure and Crohn's disease—the patient elected to undergo radical cystectomy, with final pathology confirming the absence of residual carcinoma. This case highlights the potential complementary role of BLC and tumor-informed ctDNA testing in risk stratification and counseling, while underscoring the need for further investigation to define their impact on management decisions in high-risk NMIBC with PUI. Persistent challenges include false-positive findings, limited ctDNA shedding in some tumors, and assay variability across disease stages.^12^

Cutaneous ureterostomy was selected as the urinary diversion due to the patient's Crohn's disease and the desire to avoid bowel utilization. This diversion is particularly advantageous in patients with inflammatory bowel disease, as it reduces perioperative and long-term morbidity associated with urinary exposure to bowel mucosa.^13^ Although historically less favored due to concerns regarding stomal stenosis and pouching difficulties, contemporary series have demonstrated favorable outcomes. Lockhart and colleagues reported low ureteral obstruction rates (8.2 %), preservation of renal function, and acceptable perioperative outcomes with long-term ureteral stenting.^13^

Several recent studies have validated the clinical feasibility of ctDNA monitoring in bladder cancer. Christensen et al. demonstrated that ctDNA analysis is feasible for early risk stratification, treatment monitoring, and detection of early relapse in patients with localized and locally advanced disease. Similarly, Zhang et al. reported detectable ctDNA in 52.08 % of patients with Ta-stage NMIBC, suggesting that tumor-derived DNA fragments may enter the circulation through mechanisms such as exosome release, even in the absence of disruption of the basement membrane.^14^ Detection rates and mutational concordance between ctDNA and tumor tissue were higher in T1 compared with Ta disease, supporting the concept that ctDNA levels reflect tumor invasiveness.^15^ In addition, larger tumor size has been significantly associated with higher ctDNA detection rates and greater concordance with tumor DNA, indicating a correlation between ctDNA burden and overall tumor volume.^14^

As therapeutic options for BCG-unresponsive NMIBC continue to expand, ctDNA may assume an increasingly important role in identifying patients most likely to benefit from additional intravesical therapy versus those with occult micrometastatic disease who may require definitive surgical or systemic treatment.^12^ Multiple studies, including those by Christensen et al., Lindskrog et al., Szabados et al., van Dorp et al., and Patel et al., have consistently shown that detectable ctDNA prior to cystectomy is associated with an increased risk of recurrence and inferior disease-free or overall survival ^11^^,^^16^, ^17^, ^18^, ^19^, ^20^. These findings likely reflect the presence of micrometastatic disease below the detection threshold of conventional imaging modalities. Although disease recurrence has been reported in a subset of ctDNA-negative patients, such events are substantially less frequent, highlighting both the prognostic value and current limitations of ctDNA-based assays. Ongoing refinement of ctDNA technologies is expected to further improve sensitivity and broaden their clinical applicability in urothelial carcinoma.

Finally, this case underscores important limitations of advanced diagnostic tools in the management of high-risk NMIBC. Chronic inflammatory conditions such as Crohn's disease, as well as prior exposure to intravesical BCG, may confound interpretation of enhanced cystoscopic findings. These factors emphasize the need for cautious, multidisciplinary evaluation when integrating emerging diagnostic technologies into complex clinical decision-making.

## 4 Conclusion

This case highlights the diagnostic and therapeutic challenges of managing recurrent high-risk non– NMIBC with PUI in a patient with prior pelvic radiation and Crohn's disease. The integration of advanced cystoscopic imaging with personalized ctDNA monitoring offers a more comprehensive approach to risk assessment, surveillance, and treatment planning in complex NMIBC cases, but importantly, further studies are warranted to validate the clinical utility of ctDNA in guiding management decisions for NMIBC with PUI and to evaluate its long-term impact on oncologic outcomes.

## CRediT authorship contribution statement

**Mahdi Hemmati Ghavshough:** Writing – review & editing, Writing – original draft, Visualization, Validation, Project administration, Methodology, Investigation, Data curation, Conceptualization. **Melinda Z. Fu:** Writing – review & editing, Methodology, Data curation. **Jennifer Sykes:** Writing – review & editing, Visualization. **Disha Patel:** Investigation. **Saum Ghodoussipour:** Supervision. **Vignesh T. Packiam:** Writing – review & editing, Writing – original draft, Visualization, Supervision, Project administration, Funding acquisition, Formal analysis, Data curation, Conceptualization.

## Ethics approval and consent to participate

All procedures involving human participants were conducted in accordance with the ethical standards of the institutional and national research committees and with the Declaration of Helsinki (2013 revision). Institutional Review Board (IRB) approval was obtained prior to initiation of this study.

## Consent for publication

Written informed consent was obtained from the patient for participation in this study and for publication of the case details and accompanying images. A copy of the written consent is available for review by the Editor-in-Chief of this journal upon request.

## Data availability statement

The data supporting the findings of this study are available from the corresponding author upon reasonable request. Due to patient confidentiality and institutional privacy policies, the raw clinical data are not publicly available. De-identified summary data and supporting materials can be provided upon justified academic request.

## Funding

This research did not receive any specific grant from funding agencies in the public, commercial, or not-for-profit sectors.

## Conflict of interest

The authors declare that they have no known competing financial interests or personal relationships that could have appeared to influence the work reported in this paper.
